# Agreement between questions about physical activity and sitting time, and device-based measures, used in Swedish targeted health dialogues in the context of primary health care

**DOI:** 10.1186/s13102-023-00690-8

**Published:** 2023-07-04

**Authors:** Lisbeth M. Johansson, Hans Lingfors, Marie Golsäter, Bo Rolander, Eleonor I. Fransson

**Affiliations:** 1 Unit for Research and Development in Primary Care, Futurum—Academy for Health and Care, Region Jönköping County, Jönköping, Sweden; 2 Sweden and Jönköping Academy for Improvement of Health and Welfare, School of Health and Welfare, Jönköping University, Box 1026, Jönköping, 551 11 Sweden; 3 Child Research Group, School of Health and Welfare, Jönköping University, Jönköping, Sweden; 4 Futurum—Academy for Health and Care, Region Jönköping County, Jönköping, Sweden; 5 School of Health and Welfare, Jönköping University, Box 1026, 551 11 Jönköping, Sweden

**Keywords:** Targeted health dialogue, Accelerometry, Bland-Altman plot, Evaluation, Physical activity interview form

## Abstract

**Background:**

It is important that easy-to-use measures like subjective questions about physical activity (PA) and sedentary behaviour are valid and reliable providing accurate measures, when they are used in health promotion work aiming to support people to improve their lifestyle habits such as PA. The aim of this study was to evaluate the concurrent validity of a structured interview form estimating self-reported PA and a question about sitting time used in Swedish targeted health dialogues in the context of primary health care.

**Method:**

The study was conducted in the southern part of Sweden. To evaluate concurrent validity of the interview form, time spent in moderate-to-vigorous physical activities (MVPA) and energy expenditure related to MVPA estimated by an interview form was compared with the same measures assessed by an ActiGraph GT3X-BT accelerometer. To evaluate a question about sitting time, the Swedish School of Sport and Health Sciences’ single-item question about sitting time (SED-GIH) was compared with measures from an activPAL inclinometer. Statistical analyses included deriving Bland‒Altman plots and calculating Spearman’s rank correlation coefficients.

**Result:**

Bland‒Altman plots indicated lower absolute variation in the difference between self-reported and device-based PA measures for lower PA levels, both for energy expenditure and time spent in MVPA. No systematic over- or underestimation was observed. The Spearman’s correlation coefficient between self-reported and device-based PA measures was 0.27 (p = 0.014) for time spent in MVPA and 0.26 (p = 0.022) for energy expenditure. The correlation coefficient between the single item question and device-based sitting time measures was 0.31 (p = 0.002). Sitting time was underestimated by 74% of the participants.

**Conclusion:**

The PA interview form and the SED-GIH question on sitting time may be of value in targeted health dialogues in primary health care with the intention to support sedentary and insufficiently physically active persons in increasing their physical activity and limiting their sitting time. The questionnaires are easy to use and are more cost effective than device-based measures, especially regarding population-based interventions conducted in primary health care for thousands of participants such as targeted health dialogues.

**Clinical trial registration:**

Not applicable.

**Supplementary Information:**

The online version contains supplementary material available at 10.1186/s13102-023-00690-8.

## Background

Physical activity (PA) is defined as “any bodily movement by skeletal muscles that requires energy expenditure” [1]. PA has an important impact on health and noncommunicable diseases, such as cardiovascular disease (CVD), diabetes and cancer, as well as all-cause mortality [2]. Furthermore, PA can improve mental health, quality-of-life and well-being [2]. Aerobic PA is also associated with a reduction of the detrimental effects in stress [3].

Three important dimensions of PA are intensity, frequency and duration. Intensity is the degree of energy expenditure for the PA performed in relation to time. Frequency is how often the activity is performed, and duration is how long time a person spends performing the activity [4]. The recommended PA level for adults is at least 150 to 300 min of aerobic PA of moderate intensity per week, at least 75 to 150 min of vigorous intensity per week, or a combination of these [5]. Globally, one out of four adult persons is insufficiently physically active, according to the recommended PA levels [6]. It has been observed that all PA at all intensity levels and less time spent in sedentary behaviour are associated with a lower risk of premature death [7]. From a public health perspective, it is important to reduce time spent in sedentary behaviour and increase PA of any intensity [7]. A systematic review showed that PA could reduce the health risks of sedentary behaviour [7].

According to the Swedish Health and Medical Service Act, primary health care providers have an obligation to promote healthy lifestyle habits in a population-based way, in addition to other work in primary health care [8]. The Swedish National Board of Health and Welfare and the National program for persons with unhealthy lifestyle habits describe recommended ways to advise and support persons in improving their lifestyle habits, including targeted health dialogues integrated in a primary health care context [9, 10]. The Swedish concept of targeted population-based health dialogues is a structured and systematic method to pay attention to health and risk factors for CVD as well as to identify resources to support individuals in improving their lifestyle habits. If necessary, desired follow-up visits can be provided. The Swedish concept of targeted health dialogues is used in primary health care in most counties in Sweden [10]. These health dialogues are lifestyle-oriented and aim to promote health, increase PA and decrease sedentary behaviour in the population. To facilitate a change in PA, individuals are offered support by a ‘Physical Activity on Prescription’ (PAP) [11], support at a sport centre, a step counter or a physical activity diary, and offered follow-up visits at the primary health care centre. It has previously been shown that persons participating in targeted health dialogues reported increased PA at follow up [12].

The structured PA interview form used in Swedish targeted health dialogues has previously been evaluated regarding its predictive validity concerning body mass index (BMI), the waist-hip ratio (WHR) and total cholesterol but has not yet been evaluated regarding its concurrent criterion validity [13]. Concurrent validity compares data from the PA interview form with an established method. It is therefore valuable if the questions used in the counselling situation have both predictive and concurrent validity. One method that could be used to evaluate the concurrent validity of self-reported PA is accelerometry, which relies on devices (accelerometers) recording acceleration in three dimensions. Furthermore, the accelerometers classify the intensity of the wearer’s PA [14]. The ActiGraph GT3X-BT (ActiGraph) is a triaxial accelerometer [14] with a weight of 19 g [15]. ActiGraph GT3X has previously been validated to measure PA, with an overall high inter-instrument reliability [16]. ActiGraph GT3X is also validated with regard to an overall prediction of energy expenditure for most age groups [17]. Based on established algorithms, energy expenditure can be derived from the ActiGraph in terms of kilocalorie expenditure as well as time spent in PA in different intensities [18]. In Sweden, the Swedish School of Sport and Health Sciences’ single-item question about sitting time (SED-GIH) is the only validated and recommended question about sitting time in a Swedish context [10]. This question was therefore added as an extra question in the targeted health dialogue. A uniaxial inclinometer such as activPAL can detect body positions as lying or sitting and it registers movements such as walking. It can be used for measuring sitting time [19]. The activPAL has a weight of 10 g [20]. The small size and low weight of both devices means that the participants are not disturbed by wearing the devices [21]. The activPAL has previously been validated and was found to be valid to measure body postures [19].

Self-reported estimates of PA may be affected by reporting errors and problems with interpretation or understanding of the questions [22, 23]. It might be valuable if everyone in health dialogues had device-based PA and sitting time data, but that is often not possible for practical and economic reasons [22]. It is therefore important that the questions used in targeted health dialogues regarding PA and sitting time are valid.

Hence, the aim of this study was to evaluate the concurrent validity of a structured interview form estimating self-reported PA and a question about sitting time used in Swedish targeted health dialogues in the context of primary health care.

## Methods

### Setting

The study was performed between 2017 and 2020 in a free-living condition as part of the ordinary work in six primary health care centres in the southern part of Sweden, including rural and urban areas. All inhabitants aged 40, 50, 60 and 70 years old were invited to participate in targeted health dialogues in the health care centres as part of ordinary health promotion work. At least one week before the targeted health dialogue visit, the inhabitants who had accepted the invitation to participate in the health dialogue visited the primary health care centre laboratory for blood tests included in the normal health dialogue concept. During the visit at the laboratory, the inhabitants were given information about this study and were asked to participate. Those who agreed to participate in the study received the accelerometer (ActiGraph GT3X-BT) and inclinometer (activPAL) face-to-face at the laboratory for measurement of PA and sitting time. At the targeted health dialogue, the participants returned these devices and were asked about their PA and sitting time.

Targeted health dialogues consist of a dialogue where an individual meets with a health care professional such as district nurses, nurses, physiotherapists and occupational therapists trained in the health dialogue method, health promotion and motivational interviewing [10, 24]. A pedagogically designed visual health profile, showing 13 different factors of importance for CVD is used to facilitate the targeted health dialogue. This tool is based on questionnaires about lifestyle habits (PA, food habits, tobacco use, and alcohol habits) and psychosocial factors, objective measures such as blood tests, blood pressure and anthropometric measures [12, 25] (Appendix 1). A self-reported PA interview form including questions about active transportation and moderate-to-vigorous physical activities (MVPA) during leisure-time is used. Based on the responses to these questions, time in MVPA (minutes per week), as well as energy expenditure from MVPA (kilocalories per week) can be estimated (see Appendix 2 for details) [12, 25]. Each factor in the visual health profile can be grouped into three or four levels from low to high risk for CVD. Green, yellow and red colours reinforce the visual grading, where green represents a low risk and red represents a high risk of CVD [12]. The potential to predict morbidity and mortality by means of the graded health profile has been described previously [26].

### Self-reported physical activity and sedentary behaviour

For this study, the participants were asked to recall their PA in active transportation and leisure-time physical activity (LTPA) during the past year following the normal procedure during a targeted health dialogue. If PA was reported, the participants were asked to describe what kind of LTPAs they performed and for how long time they performed the activities in minutes during a normal week across the four different seasons spring, summer, autumn and winter. The health care professional conducting the targeted health dialogue noted the participants’ answers using the PA interview form, with the possibility to ask elaborating questions. The time spent in different physical activities was multiplied by different intensity factors based on the original Minnesota Leisure Time Physical Activity Questionnaire and Passmore and Durnin method [27, 28]. From the algorithm in the PA interview form, the total energy expenditure for all MVPAs conducted during leisure-time was calculated expressed as kilocalories per week. In addition, the participants’ commuting habits were assessed. If they commuted using some kind of physically active transportation method the total energy expenditure expressed as kilocalories per week was calculated considering the type of transportation, season, distance to work and frequencies in a way similar to that for LTPA. The time and energy expenditure spent in MVPAs during leisure-time and active transportation, respectively, were then summed to obtain the total average time (minutes per week) and energy expenditure (kilocalories per week). Appendix 2 shows how the PA exercise score was calculated [25]. The score of energy expenditure in terms of kilocalorie expenditure per week during active transport and MVPA during leisure-time was then reported in the Health Curve (Appendix 1).

One question concerning sitting time was also asked. The SED-GIH *‘How much time do you sit during a normal day excluding sleep?*’ was asked, with seven categorical answer choices: *‘Virtually all day’*, *‘13–15 hours’, ’10–12 h’, ‘7–9 h’, ‘4–6 h’, ‘1–3 hours’* and *‘never’* [29]. The participants answered the question in a paper questionnaire.

### Device-based measurement of physical activity and sedentary behaviour

**Fig. 1:**
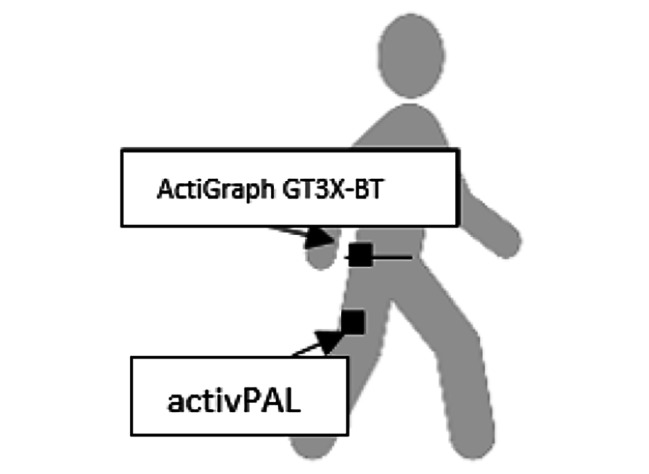
Body position for ActiGraph GT3X-BT and activPAL

The devices were distributed face-to-face to the study participants in the study by the laboratory staff at the primary health care centre. They were instructed by the laboratory staff to wear an ActiGraph GT3X-BT accelerometer from ActiGraph LCC Pensacola, FL, USA (Fig. 1). They also wore an activPAL inclinometer (PAL Technologies LTD. Glasgow, Scotland) (Fig. 1). They were instructed to wear these devices for 24 h a day for seven consecutive days to cover all weekly activities before the health dialogue visit. At least five days of wear time for both devices was required as a minimum for analysis [30].

The participants wore the ActiGraph on the right hip (Fig. 1) [14]. The ActiGraph was a triaxial accelerometer, that detects acceleration in the vertical, medio-lateral and antero-posterior axes [14]. The activPAL was a uniaxial inclinometer that detects body positions such as standing, lying and sitting, and it registers movements such as walking [19]. The activPAL was covered by a waterproofed tube and Tegaderm transparent dressing and placed on the frontal midline of the right thigh (Fig. 1) [19]. The participants were asked to keep a logbook during the days they wore the devices. They noted the time they got up in the morning, time taken for transport to work, time of returning from work and breaks during working hours, time of starting and ending PA and times when the ActiGraph was, temporary removed e.g., when taking a shower.

### Data analysis of physical activity and sitting time

Time spent in MVPA (minutes per week) as well as energy expenditure (kilocalories per week) based on MVPA were analysed both from self-reported data from the logbook and the PA interview form and compared with data from the ActiGraph and analysed in the ActiLife 6.13.4 program (ActiGraph LCC Pensacola, FL, USA) [15]. Time spent sitting was also analysed by using self-reported data from the SED-GIH and data from the activPAL and analysed with PAL software Suite version 7 (PAL Technologies LTD. Glasgow, Scotland) [31]. The data from the ActiGraph were downloaded in an epoch length of 10 s and with data sampling of 30 Hz. In the analysis in the ActiLife program, the algorithm from Choi 2011 [32] was chosen for wear time validation together with a minimum of at least 540 min of wear time per day for at least five days. Of these five days, at least three days had to be weekdays and one day had to be a weekend day [30, 33]. Nonwear time, which exceeded more than 10 min, was also excluded from the analysis [32]. If any participant had forgotten to write down their bedtime in the evening and awakening time in the morning, it was determined by using the activPAL data. Sleeping periods were excluded from the analysis. For the analysis, data from the participant’s logbook for LTPA and active transportation were imported into the ActiLife program´s log-diary. Data were scored according to the cut points for the intensity of PA with sedentary behaviour and energy expenditure defined in the program ActiLife according to Freedson [18]. Indirect calorimetry with walking and running as tested activities was used to create this algorithm and it also includes BMI when transforming accelerometer activity counts into energy expenditure. The cut points were as follows: 0-100 counts per minute was defined as sedentary behaviour, ≤ 1951 counts per minute was defined as light intensity activity, 1952–5724 counts per minute was defined as moderate intensity activity, 5725–9498 counts per minute was defined as vigorous intensity activity and ≥ 9499 counts per minutes was defined as very vigorous intensity activity [18]. Time spent in MVPA was measured each day, and energy expenditure in physical activities per week outside work was included for the season that the participants wore the devices. The duration of MVPA water training noted in the participants’ logbooks was added to the data measured by the ActiGraph, as the ActiGraph was not used in the water. In the analysis of energy expenditure, water training was excluded since we did not have data concerning energy expenditure from the ActiGraph. To make self-reported PA regarding transport comparable with the time (in minutes) presented for self-reported LTPA for each season, the amount of active transport had to be converted to minutes (for details see Appendix 2). For each season, the amount of active transport by bicycling were divided by four, with the anticipation it will take four minutes to cycle one kilometre. Correspondingly, the amount of active transport by brisk walking were divided by ten with the anticipation it will take 10 min to walk one kilometre.

For those wearing the devices for only five or six days, data for time spent in MVPA and energy expenditure were multiplied by 7/5 (= 1.4) or 7/6 (= 1.17), respectively, to make the time comparable with seven days of wear time, since self-reported PA from the interview form was based on average PA during seven days. For time spent in MVPA and energy expenditure 19 participants had six days and 19 participants had ActiGraph recordings for five days. In the analysis, we only included transport PA for the season in which the participants wore the device.

The body position and sitting time were measured by the activPAL, which recorded data from the orientation of the thigh at a sampling rate of 20 Hz. Data were analysed using activPAL professional software, PAL Technologies LTD. version 7.3.28 Glasgow, Scotland [31].

The sitting time for the days the participants wore the activPAL for the whole day was summed and then divided by the number of days they wore the devices to obtain the average sitting time per day. The daytime sitting time was calculated by using the data from the activPAL. The result was categorized into the same categorical answer options as in SED-GIH [29].

### Statistical analyses

Descriptive analyses of numbers and proportions in each group were conducted concerning the background characteristics of the participants. As part of the calculations for the Bland‒Altman plots, the mean difference between the device-based measures and self-reported PA estimates were calculated and one-sample t tests of the difference were conducted. Agreement between MVPA based on accelerometry and MVPA based on self-reported data was evaluated by constructing Bland‒Altman plots [34] regarding time (minutes per week) and energy expenditure (kilocalories per week) spent in MVPA, both as continuous variables. The correlation between accelerometer MVPA data and self-reported MVPA data was calculated with Spearman’s rank correlation coefficient (Spearman’s rho) with a p value less than 0.05 considered as statistically significant.

The correlation between device-based measurements by activPAL and self-reported categorical SED-GIH during the day was evaluated by Spearman’s rank correlation coefficient. The proportion of people under- or overestimating their sitting time was calculated by taking the sum of the proportion of individuals with deviations from the diagonal in a cross-tabulation of categories with device-based measured and self-reported estimated sitting time.

IBM SPSS statistic version 27.0 IBM Corp, Armonk, New York, USA) was used in the analysis.

### Ethical issues

The study received ethical approval from the Regional Ethical Review Board in Linköping (#2016 367 − 31; #2017 65 − 32; #2019 05056). The study was conducted in accordance with the Declaration of Helsinki. Prior to participating all participants were properly informed about all study procedures and gave written informed consent to participate in the study.

## Results

### Participants

**Fig. 2:**
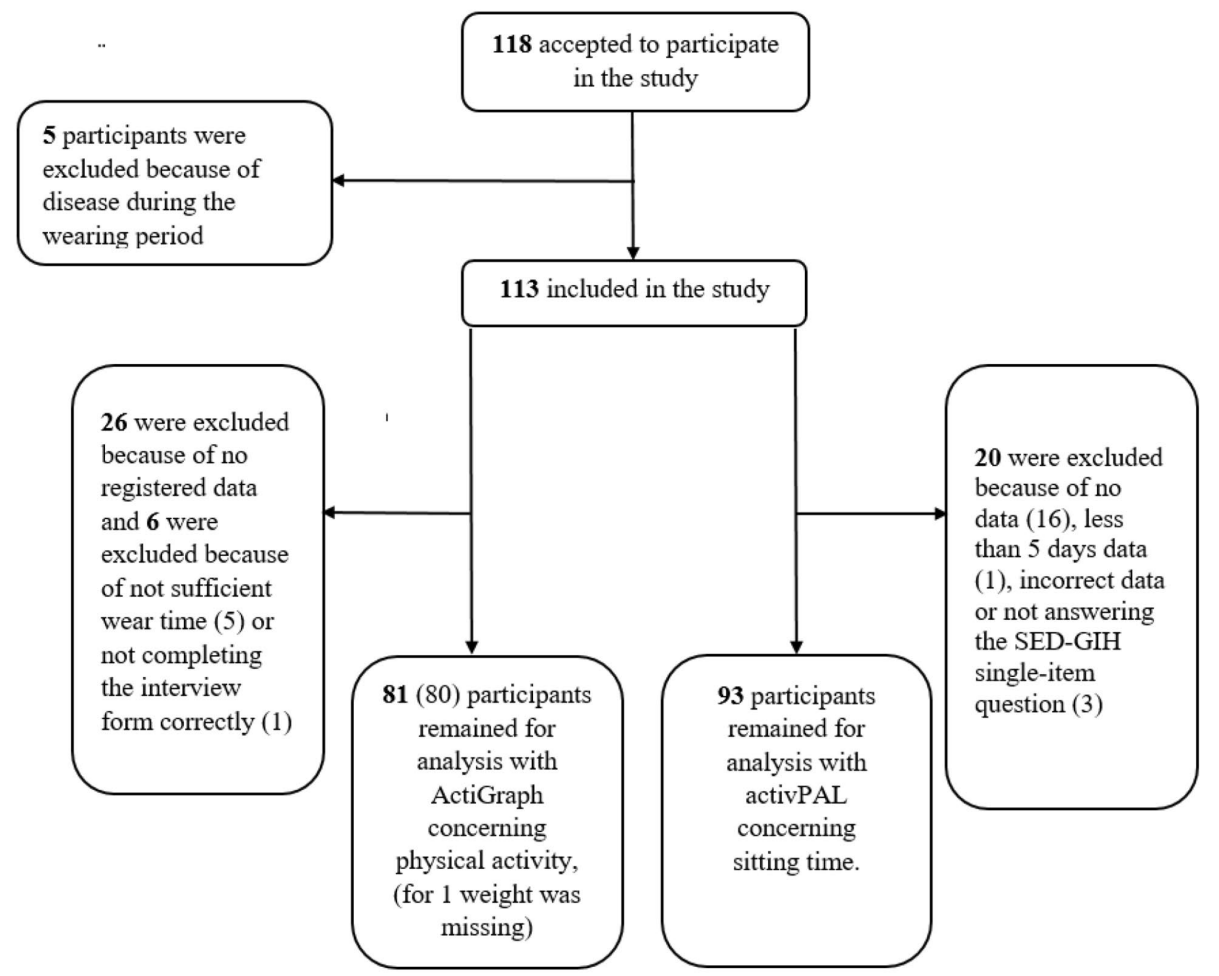
Flow chart for participants with data available for analysis concerning active transport, leisure time moderate- to vigorous physical activity and sitting time

The study invitation was accepted by 118 persons (Fig. 2). In total, 113 individuals participated, of whom 81 had device-based PA data for time spent in MVPA (minutes per week), and 80 had energy expenditure data (kilocalories per week) measured by the ActiGraph GT3X-BT, while 93 had device-based data for sitting time, measured by the activPAL.

Table 1 shows the background characteristics of the participants. The mean (± SD) age was 56.3 ± 11.0 years.

**Table 1:** Background characteristics of the 113 participants

		n	%
Sex	Male	47	41.6
	Female	66	58.4
Age	40 years	20	17.7
	50 years	36	31.9
	60 years	23	20,4
	70 years	34	30.1
Smoking habits	Non smokers	105	92.9
	Smokers	6	5.3
	Not specified	2	1.8
BMI	0–25 kg/m^2^	55	49.5
	25.1–30 kg/m^2^	42	32.2
	Over 30 kg/m^2^	14	12.4
	Not specified	2	1.8
Self-rated economy situation	Economic problem	12	10.7
	No economic problem	98	86.7
	Not specified	3	2.7
Educational level	Short education (Nine years or less)	15	13.3
	Intermediate education (Nine to 12 years)	47	41.6
	Long education (University level)	33	29.2
	Not specified	18	15.9
Season the device was worn	Spring Summer Autumn Winter	49 7 29 28	43.4 6.2 25.7 24.8

**Fig. 3:**
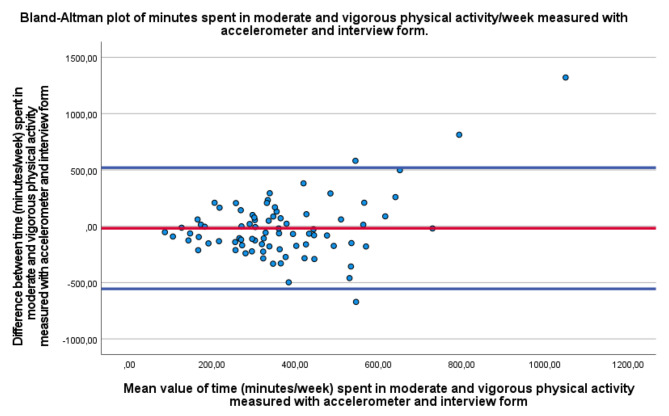
The Bland‒Altman plot illustrates the closeness of agreement for time spent in MVPA for active transport and LTPA in minutes per week measured with the accelerometer and as estimated from the PA interview form. The y-axis shows the difference between the accelerometer data for time spent in MVPA for active transport and LTPA measured with the accelerometer and the estimated self-reported time spent in MVPA for active transport and LTPA from the PA interview form. The x-axis shows the mean value of time spent in MVPA for active transport, and LTPA measured with the accelerometer and the self-reported time from the PA interview form. The limits of agreement enclose observations within ± 2 standard deviations, which means that approximately 95% of the cases were between the upper and lower horizontal lines

The results of Bland‒Altman plots presented in Fig. 3 show a mean value of 376 ± 163 min per week spent in MVPA when measured by ActiGraph compared with 359 ± 252 min per week when based on PA from the interview form. This is a mean difference value of -17 ± 274 min per week, t=-0.57, degrees of freedom (df) = 80, p value = 0.57. The 95% limits of agreement ranged between − 520 and 555 min in MVPA per week.

**Fig. 4:**
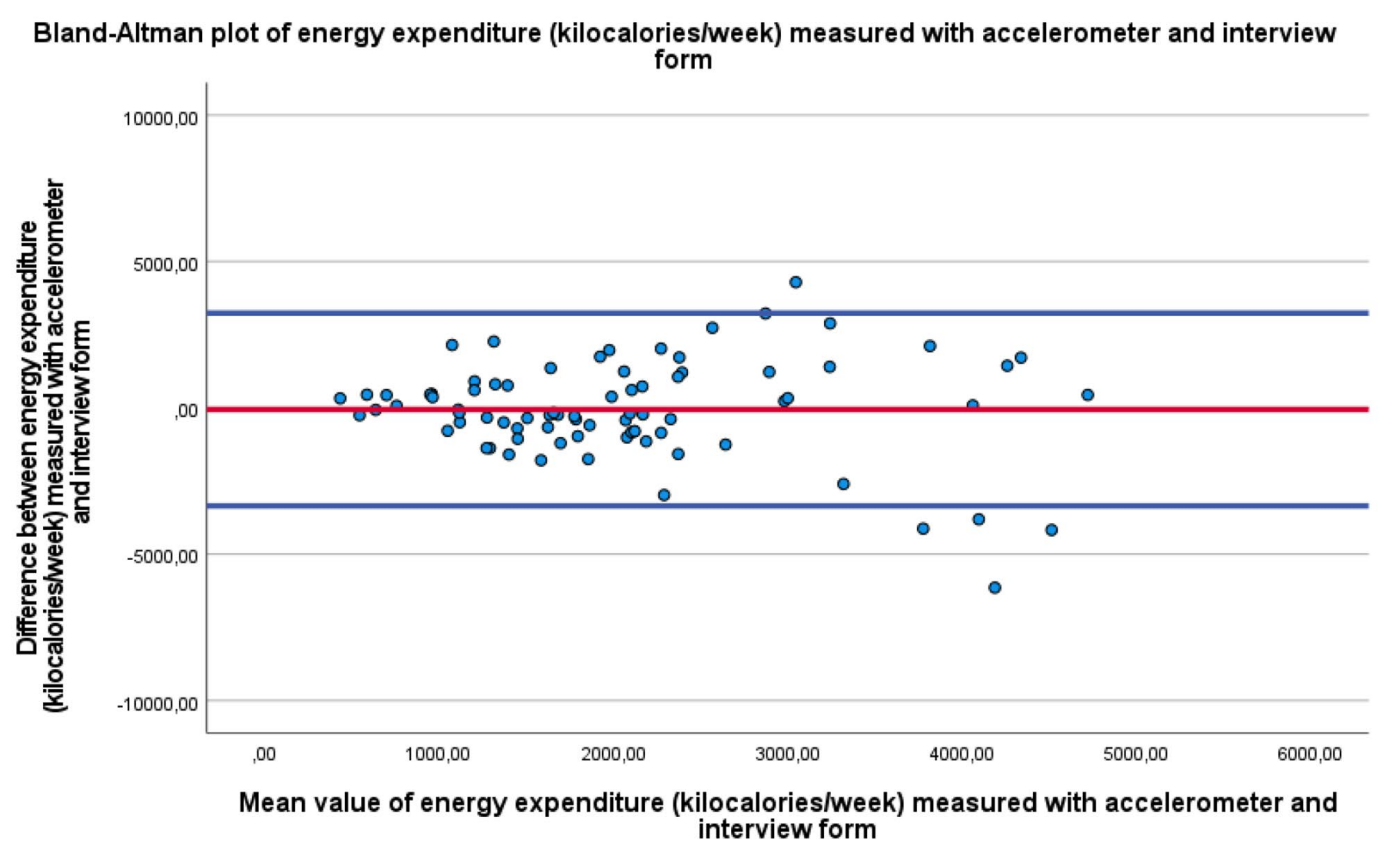
The Bland‒Altman plot illustrates the closeness of agreement for energy expenditure, in terms of kilocalories per week, for MVPA in active transport and LTPA. The y-axis shows the difference between the accelerometer data for energy expenditure (in kilocalories per week) for MVPA in active transport and LTPA measured with the accelerometer and the self-reported energy expenditure in terms of kilocalories per week for MVPA in active transport and LTPA from the PA interview form. The x-axis shows the mean value of the energy expenditure for active transport and LTPA spent in MVPA measured with the accelerometer, and the self-reported energy expenditure in terms of kilocalories per week for MVPA in active transport and leisure time physical activity from the PA interview form. The limits of agreement enclosed observations within ± 2 standard deviations, which means that approximately 95% of the cases were between the upper and lower horizontal lines

For energy expenditure (kilocalories per week) spent in MVPA during active transport and LTPA, as shown in the Bland‒Altman plot in Fig. 4, the mean value measured with the ActiGraph was 2096 ± 1390 kilocalories per week, and for self-reported energy expenditure, the mean value was 2040 ± 237 kilocalories per week. The difference in the mean value was − 56 ± 1680 kilocalories per week, t=-2.96, df = 79, p value = 0.70, with the 95% limits of agreements ranging between − 3988 and 3666 kilocalories per week.

Spearman’s correlation coefficient between time spent in MVPA (minutes per week) estimated from self-reported data and ActiGraph data is modest (Spearman’s rho p = 0.014) (Table 2). The correlation between self-reported data on sitting time and data from the activPAL is moderate (rho = 0.31, p = 0.002) (Table 2).

**Table 2:** Correlation between objectively measured data and self-reported data for time spent in MVPA per week, energy expenditure per week and sitting time

	n	Spearman’s rho	P value
Time spent in MVPA (minutes/week). ActiGraph data compared to self-reported MVPA.	81	0.27	0.014
Energy expenditure (kcal/week). ActiGraph data compared to self-reported energy expenditure.	80	0.26	0.022
Sitting time (hours/day). ActivPAL data compared with self-reported categorical data.	93	0.31	0.002

Sitting time analysed with the SED-GIH was underestimated by 74% of the participants in comparison to sitting time measured with the activPAL. The participants’ self-reported sitting time and sitting time derived from the activPAL by time category are presented in Table 3.

**Table 3:** Distribution of participants (n = 93) sitting time from questionnaire and activPAL.

N = 93	Self-reported sitting time from the SED-GIH question.	Sitting time from the activPAL data
Virtually all day	0	0
13–15 h	2	0
10–12 h	6	24
7–9 h	16	58
4–6 h	45	10
1–3 h	24	1
never	0	0

The PA interview form showed that brisk walking was the most common activity in all seasons. Brisk walking constituted 39% of all reported activities in the spring season, 29% in the summer season, 45% in the autumn season, and 47% in the winter season.

## Discussion

The results of this population-based study show a rather weak but acceptable agreement for PA measured with the accelerometer and self-reported PA calculated from the interview form, both for time spent in MVPA and energy expenditure. Bland‒Altman plots indicated lower absolute variation for persons with a lower PA level both concerning time spent in MVPA and for energy expenditure. It should be noted that other studies have shown that accelerometry has difficulty detecting activities at high intensity levels by an underestimation of vigorous PA partly related to a band pass filter in the ActiGraph [35].

The analyses showed no systematic over- or underestimation of self-reported time spent in MVPA or energy expenditure estimated by the structured PA interview form. This is in contrast to other studies that have shown an overestimation of self-reported PA [7]. This is important information for clinical practice in that the participants do not systematically over- or underestimate their PA. However, the limits of agreement in this study enclosed a broad interval, indicating that the self-reported PA estimates were rather imprecise. Self-reported measures of PA are known to be imprecise, as observed in other studies [7, 22]. This is worth remembering when interpreting self-reported PA and advising persons in the clinical setting.

This study showed that the PA interview form may be acceptable to use during targeted health dialogues promoting PA, especially for people reporting low, medium and high levels of PA, but less so for those reporting very high levels of PA. This may be a concern with the wide limits of agreement between device-based measured and self-reported PA. However, the analyses showed the lowest absolute variation among persons with the lowest PA level. This means that the PA interview form may be more useful for those with lower levels of PA. The persons who will have the greatest benefits from this kind of support are those who are inactive or insufficiently physically active and need support to start performing some PA or increase their PA. Furthermore, self-reported PA estimates are cost-effective and valuable in promoting PA in health care environments [22].

Spearman’s rho was 0.27 for the time spent in MVPA per week and 0.26 for energy expenditure between device-based and self-reported data. The Spearman’s rho correlations of 0.27 and 0.26 can be considered modest, according to Muijs [36]. These modest correlations are similar to those found in another Swedish study comparing MVPA measured with accelerometers and self-reported MVPA [37]. The results are also in line with a systematic review analysing correlations between accelerometer data and self-reported PA [38]. In this review comprising 41 studies, only one study reported a correlation coefficient >0.50 between self-reported PA and accelerometer data. In the other 40 studies, the correlation coefficients varied between 0.25 and 0.37 [38]. Still, according to Sattler [22], self-reported PA has provided valuable information and insights into PA and health over the decades. Self-reported PA has supported the global guidelines of PA [22]. PA is difficult to measure because it consists of many different types of PA, such as active transport and LTPA.

We observed a clear pattern in which sitting time was underestimated by a majority of the participants. A similar result was reported in another Swedish study using the Swedish SED GIH [29] as well as in a meta-analysis [39]. Spearman’s rho of 0.31 for sitting time in our study was also in line with the Swedish study [29]. This correlation could be considered moderate, according to Muijs [36]. The tendency to underestimate the sitting time is worth taking into consideration when interpreting self-reported sitting time and advising persons to decrease their sitting time and replace it with PA in the clinical setting. Replacing sitting time with PA is likely to decrease the risk of premature death and death from cardiovascular diseases [7, 40].

Ideally, every participant in the targeted health dialogues should wear advanced devices to measure MVPA and sitting time before health dialogue visits, but this is not possible in population-based targeted health dialogues with thousands of participants for practical and economic reasons. Therefore, it is common to use different kinds of questionnaires in larger health promoting initiatives [22]. However, the development of electronic questionnaires and different types of devices has brought new opportunities to this research area [22]. As this research field is rapidly advancing, the associations between PA as well as sitting time, and various health-related outcomes may turn out to be even stronger when measures of PA and sedentary behaviour are device-based instead of self-reported [41, 42]. However, today, we have to accept the validity of questionnaires, as observed in our study and other studies [38] and as is discussed in a recent Swedish study [43].

It should be noted that the mean values were over 300 min per week for time spent in MVPA and over 2000 kilocalories per week for energy expenditure when measured with the ActiGraphs as well as when estimated with the PA interview form. This is well above the PA level recommended by World Health Organization (WHO) [2]. A similar pattern was also observed in another study [37]. One possible explanation for this may be that people with higher MVPA are perhaps more likely to choose to participate in a study with devices that measure PA. Their PA patterns may be different in comparison to those of persons with lower PA. It could also be that the awareness of wearing an activity device may stimulate to more activities than usual, and participants may increase their PA during the period in which they wear the devices [41]. On the other hand, this effect may perhaps not be so pronounced with ActiGraphs and the activPAL because participants do not receive any PA feedback results compared to, for example, pedometers [42].

### Strengths and limitations

The use of both an accelerometer and an inclinometer for concurrent validity was a strength of our study, as they have different strengths and limitations. Accelerometers are useful for measuring PA but are not as useful for measuring sedentary behaviour, such as sitting time, as they do not differentiate body position [44]. The inclinometer is, on the other hand, valid for distinguishing between sedentary and standing postures [19].

A strength of this study was that the devices were distributed to the participants face-to-face at the primary health care centre laboratory. A face-to-face distribution of accelerometers has been shown to be feasible in smaller studies [30]. Furthermore, this validity study was conducted in a real-life situation, which is a strength to the study. Another strength is that the primary health care centres that participated in the study were in both urban and rural areas. The participants in this study were representative regarding BMI, tobacco use, and level of education compared to those who were invited to targeted health dialogues in the county where the study was conducted [45]. Furthermore, the number of participants in this study was fairly high. For example, in a systematic review article, it was proposed that the number of participants should exceed 50 in this type of study. In our study, the number of participants exceeded 50 in all analyses [38]. A limitation of the study is that we did not have full information about the number of invited persons to provide complete participation rates.

Another strength of this study is that we used the ActiLife log-book to identify both active transport PA and LTPA, as these are also estimated in the PA interview form. Furthermore, it was a strength that we matched the season in which the participants wore the devices with the same season reported in the PA interview form.

A limitation of using accelerometry is that it may not measure some types of activities accurately, such as activities that mainly involve the upper body [30] and strength training [41]. This can affect the agreement between device-based measured and self-reported PA.

Another weakness is that the participants came from only one county in southern Sweden. This may affect the generalization due to different PA preferences in other counties, for example, counties with more countryside areas, counties with long commutes to work and large cities.

A weakness could be, that we in this study used the algorithm of Freedson 1998 to calculate energy expenditure from accelerometer data. This algorithm includes BMI and transforms accelerometer activity counts into energy expenditure by indirect calorimetry with walking and running as tested activities [18]. An overview of objective measures revealed that even if there are concerns with transforming accelerometer counts into energy expenditure, there is a good linear relationship between energy expenditure for walking and accelerometer count-based energy expenditure [46]. In our study, brisk walking was the most common activity.

## Conclusions

The results indicate a better agreement for lower levels of time spent in MVPA and energy expenditure. No systematic over- or underestimation was observed. The correlations between device-based measured and self-reported PA were modest. Sitting time was generally underestimated. The results are in line with previous studies. Being aware of these limitations, the PA interview form and the SED-GIH may be of value in targeted health dialogues with the intention to support sedentary and insufficiently physically active persons in limiting their sitting time and increasing their physical activity. The questionnaires are easy to use and are more cost effective than device-based measures, especially in regard to population-based interventions conducted in primary health care settings with thousands of participants, such as targeted health dialogues.

## Electronic supplementary material

Below is the link to the electronic supplementary material.

Supplementary Material 1

Supplementary Material 2

## Data Availability

The datasets used during the current study are available from the corresponding author on reasonable request.

## List of abbreviations

ActiGraph: -ActiGraph GT3X-BT
CVD: Cardiovascular disease
df: Degrees of freedom
LTPA: Leisure-time physical activity
MVPA: Moderate-to-vigorous physical activity
PA: Physical activity
PAP: Physical activity on prescription
SD: standard deviation
SED-GIH: Swedish School of Sport and Health Sciences’ single-item question about sitting time
Spearman’s rho: Spearman’s rank correlation coefficient
WHO: World Health Organisation
