# Supplementary material for: Agreement between questions about physical activity and sitting time, and device-based measures, used in Swedish targeted health dialogues in the context of primary health care
Source: BMC Sports Sci Med Rehabil. 2023 Jul 4;15:76. doi: 10.1186/s13102-023-00690-8 (PMC10318802; doi:10.1186/s13102-023-00690-8)
Supplement: Supplementary file 1 — Supplementary Material 1 [file 13102_2023_690_MOESM1_ESM.pdf]

Appendix 1

Health Curve

Sex |__| Age |__|__| Group |__|__| P Nr |__|__|__|__|__|__| - |__|__|__|__|

Nr |__|__| |__|__| |__|__|__|__|__| Visit Nr |__|__|
C PCC Nr

DATE |__|__|__|__|__|__|__|__| .... |__|__|
Examiner

Item	Entry	Low risk ← 1	2	3	→ High risk 4
**1 Physical Activity,** kcal/week	|__|__|__|__|	> 2000	1000 - 2000	500 - 999	< 500
**2 Food,** Fat |__|__|__| Fibre |__|__|	Dietary points |__|__|	3 - 5	6 - 8	9 - 11	
**3 Alcohol,** units (glasses)/week	|__|__|__|	♂ ≤ 6<br>♀ ≤ 4	7 - 13<br>5 - 8	14 - 18<br>9 - 12	> 18<br>> 12
**4 Tobacco,**	Cig/day |__|__| Snuffboxes/week |__|__|	0/ex	snuff	1 - 9	> 9
**5 Life Situation,**	|__|	< 2	2 - 3	4 - 5	
**6 Mental Distress,**	|__|	< 3	3 - 4	5 - 7	
**7 Heredity, Diabetes,**	|__|	0	1	≥ 2	
**8 Heredity, CVD,**	Father |__|__| Mother |__|__|	≥ 70<br>≥ 75	55 - 69<br>60 - 74	45 - 54<br>50 - 59	< 45<br>< 50
**9 Body Mass Index,** kg/m^2	|__|__|__|	♂< 27<br>♀< 29	27 – 36,9<br>29 – 38,9	≥ 37<br>≥ 39	
**10 Waist-Hip-Ratio**	|__|__|__|	♂< 0,90<br>♀< 0,78	0,90 - 0,94<br>0,78 - 0,82	0,95 - 0,99<br>0,83 - 0,87	≥ 1,00<br>≥ 0,88
**11 Blood Pressure,** mm Hg	|__|__|__| - |__|__|__|	< 140<br>< 90	140 – 169<br>90 - 104	170 – 199<br>105 - 114	≥ 200<br>≥ 115
**12 Cholesterol,** mmol/l	|__|__|__|__|	♂< 5,00<br>♀< 5,00	5,00 - 6,49<br>5,00 - 7,09	6,50 - 9,00<br>7,10 - 9,00	> 9,00<br>> 9,00
**13 Chronic Disease**..	|__|__|__|__|				

14 Weight, kg .... |__|__|__|__|

15 Height, metre .... |__|__|__|

16 Waist, cm .... |__|__|__|

17 Hip, cm .... |__|__|__|

18 LDL, mmol/l .... |__|__|__|

19 HDL, mmol/l .... |__|__|__|

20 Triglyc, mmol/l ...... |__|__|__|__|

21 P-glucose, ... |__|__|__|

22 γ-GT, μkat/l .... |__|__|__|

23 Hb, g/l .... |__|__|__|

24 fP-Glucose, g/l .... |__|__|__|

25 .... |__|__|__|__|

Follow-up? |__|
No=1 Yes = 3

To improve 1......... |__|__|

To improve 2......... |__|__|

To improve 3......... |__|__|
FA=1, Diet=2, Alcohol=3, Tobacco=4, Life situation = 5, Distress=6, BMI=9, WHR=10, BP=11, Cholesterol=12

How 1 .... |__|

How 2 .... |__|

How 3 .... |__|
FA=1, Diet=2, Alcohol=3, Tobacco=4, Life situation=5, Distress=6, Others=7

Support by 1 .... |__|

Support by 2 .... |__|

Support by 3 .... |__|
No one=1, Nurse=2, Group=3, Doctor=4, Other (e.g. physiotherapist, psychologist, dietician etc.)=5

© Department of Research and Development, Futurum, Jonkoping
