# Supplementary material for: Agreement between questions about physical activity and sitting time, and device-based measures, used in Swedish targeted health dialogues in the context of primary health care
Source: BMC Sports Sci Med Rehabil. 2023 Jul 4;15:76. doi: 10.1186/s13102-023-00690-8 (PMC10318802; doi:10.1186/s13102-023-00690-8)
Supplement: Supplementary file 2 — Supplementary Material 2 [file 13102_2023_690_MOESM2_ESM.docx]

Additional file 2 Appendix 2


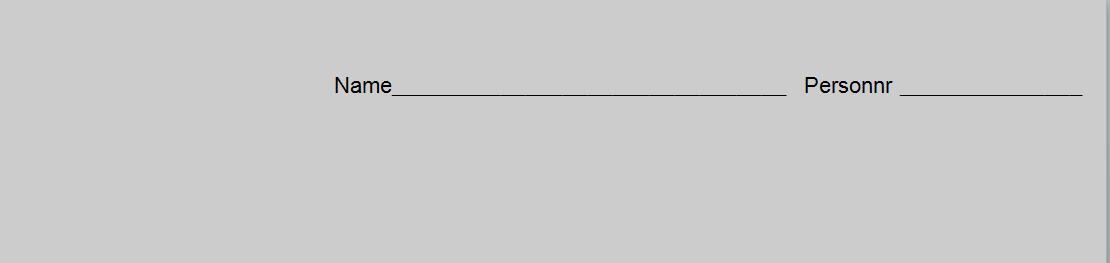


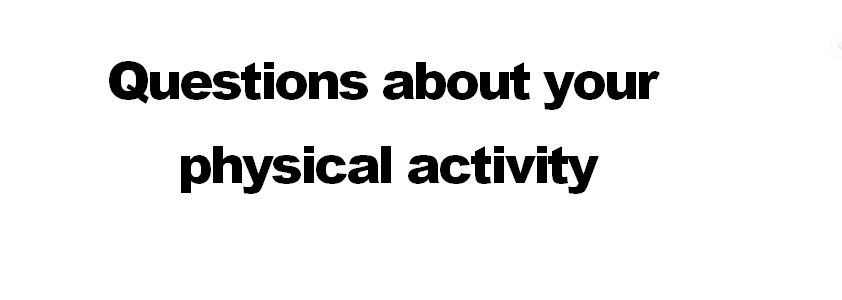


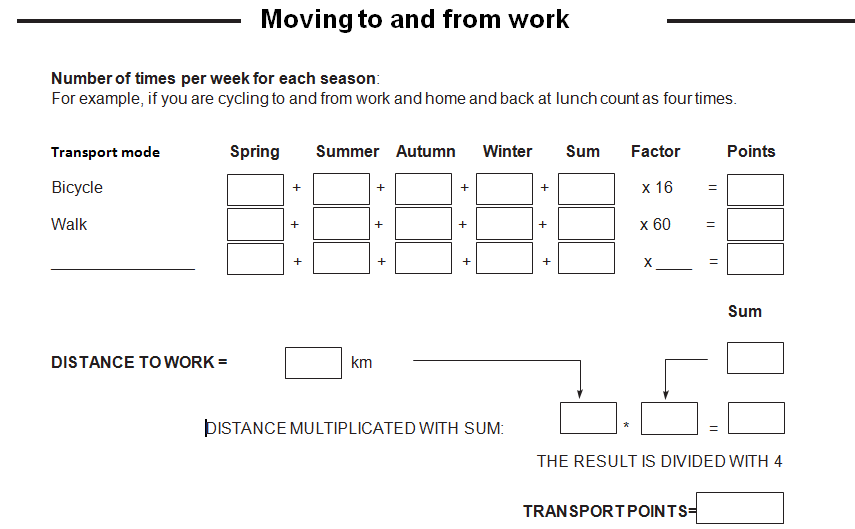


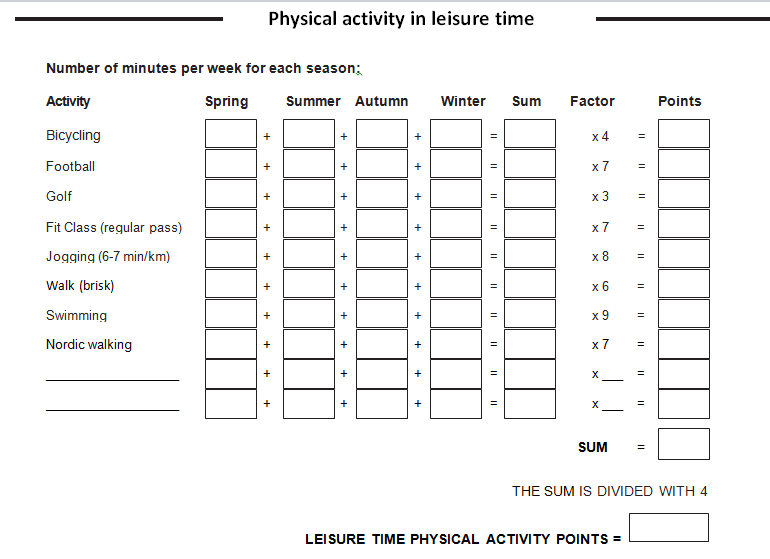


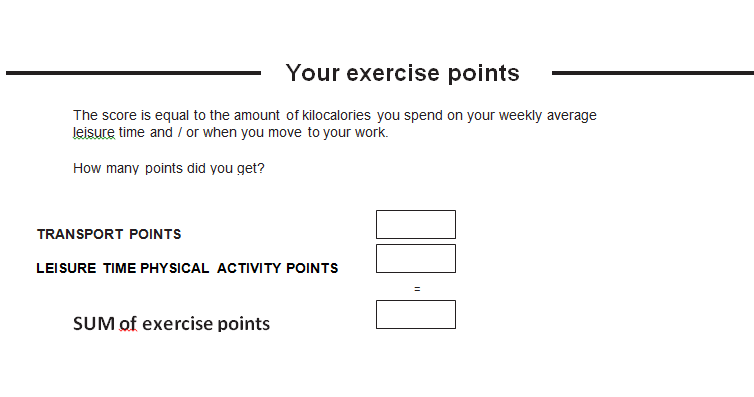


500 kcal You have too little physical activity! If you want to increase your physical activity, try to to start with 5 minutes daily and then try to increase to at least 30 minutes each day.

500–999 kcal You do quite a lot of physical activity, but can achieve more health benefits if you increase your physical activity. Consider whether you can add some extra physical activity!

1000–2000 kcal Good! You are probably more physically active than the most. Keep on with your activities!

> 2000 kcal You are among those whom have a lot of physical activity and may have made it to a lifestyle. Keep on with this!

© Department of Research and Development, Futurum, Jonkoping
